# Alpha-synuclein structure and Parkinson’s disease – lessons and emerging principles

**DOI:** 10.1186/s13024-019-0329-1

**Published:** 2019-07-22

**Authors:** Richard M. Meade, David P. Fairlie, Jody M. Mason

**Affiliations:** 1 Department of Biology & Biochemistry, University of Bath, Claverton Down, Bath, BA2 7AY UK; 2 Division of Chemistry and Structural Biology, Australian Research Council Centre of Excellence in Advanced Molecular Imaging, Institute for Molecular Bioscience, The University of Queensland, Brisbane, Queensland 4072 Australia

**Keywords:** Alpha-synuclein, Amyloid, Oligomers, Parkinson’s disease, Protein-protein interactions, CryoEM

## Abstract

Alpha-synuclein (αS) is the major constituent of Lewy bodies and a pathogenic hallmark of all synucleinopathathies, including Parkinson’s disease (PD), dementia with Lewy bodies (DLB), and multiple system atrophy (MSA). All diseases are determined by αS aggregate deposition but can be separated into distinct pathological phenotypes and diagnostic criteria. Here we attempt to reinterpret the literature, particularly in terms of how αS structure may relate to pathology. We do so in the context of a rapidly evolving field, taking into account newly revealed structural information on both native and pathogenic forms of the αS protein, including recent solid state NMR and cryoEM fibril structures. We discuss how these new findings impact on current understanding of αS and PD, and where this information may direct the field.

## Background

Parkinson’s Disease (PD) is a progressive neurodegenerative disease, which accounts for approximately 15% of all dementia cases [1], and is the second most common form of neurodegeneration to Alzheimer’s disease [2]. The disease has a mean onset of 55 years old and exhibits both physical and neuropsychiatric symptoms. The physical symptoms include slow imprecise movements (bradykinesia), tremors at rest, rigidity, facial paucity (hypomimia), shuffling gait, difficulty walking, freezing and postural instability [2]. The neuropsychiatric symptoms, which occur at later stages of the disease, manifest as cognitive defects, specifically slowness, disrupted sleep, and sensory disturbances, leading to suffers becoming passive and withdrawn [2].

PD is thought to be largely caused by the death of dopaminergic neurons in the *substantia nigra pars compacta*, located in the basal ganglia of the brain. This region of the brain is involved in coordinating movement, sending signals down the spinal cord to control muscle contraction, meaning that damage to this region can compromise signalling, leading to the physical symptoms of PD.

A wide range of both environmental and genetic risk factors have been implicated in the pathogenesis of PD [3]. Environmental risk factors include pesticides (specifically organochlorines) [4] and ambient air pollution [5]. Interestingly, tobacco [6], coffee [7], black tea [8], and a few pharmaceuticals including statins [9], calcium channel blockers [10] and ibuprofen [11], have shown some evidence of neuroprotective properties in a few studies. Autosomal dominant risk factors implicated with PD were first found in the SNCA gene that encodes αS, the primary component of Lewy bodies that are characteristic of all synucleinopathies. This will be discussed in detail and is the main focus of this review. It is worth noting that there are a number of other autosomal dominant and recessive risk factors implicated in PD, some of which occur upstream of the toxicity caused by αS. Other autosomal dominant mutations are found in the Leucine rich repeat Kinase 2 (LRRK2) domain, accounting for 4% of familial PD [12], in the vascular protein sorting 35 (VPS35) gene [13], accounting for 1% of familial PD and in the CHCHD2 [14] and eIF4G1 [15] genes. Recessive genes implicated in familial PD are Parkin [16], PTEN-induced putative kinase 1 (PINK1) [17], and Daisuke-Junko-1 (DJ1) [18] genes. These genes are upstream mutations which appear to increase αS toxicity, suggesting that further advances in understanding αS structure and function may be crucial to understanding and ultimately treating PD.

PD is strongly associated with the appearance of dopaminergic neuronal cytoplasmic inclusions called Lewy bodies. These are the leading pathogenic hallmarks in brain biopsies of PD patients, and are not present in healthy individuals. In 1997 Lewy body inclusions were shown to contain aggregates of αS [19], a 140 amino acid protein which has consequently been implicated as the likely cause of familial PD [20–22]. Further evidence is provided by the fact that duplication, triplication and autosomal dominant missense mutations in the SNCA gene lead to early onset forms of PD. It is now believed that the misfolding and subsequent aggregation of αS is a primary cause of dopaminergic degradation in PD. This is confounded by a rapidly ageing global population, correlating with an increasing number of sporadic cases of PD. In the United Kingdom alone it is believed that about 0.2% of the population are living with PD, affecting an estimated 127,000 people, and currently costing the NHS approximately £212 million per year [23]. This highlights the importance of discovering new methods to diagnose, treat and especially prevent neurodegeneration associated with PD and related synucleinopathies, and to better understand their pathogenesis. Effective strategies for preventing or reversing αS aggregation and neurotoxicity are urgently needed to avoid an exponential increase in disease with an ageing population. Recent solid state NMR and cryoEM fibril structures have brought new structural insights to the folding and formation of both native and pathogenic conformations of the αS protein [24–27].

### α-Synuclein: native structure and function

**Fig. 1:**
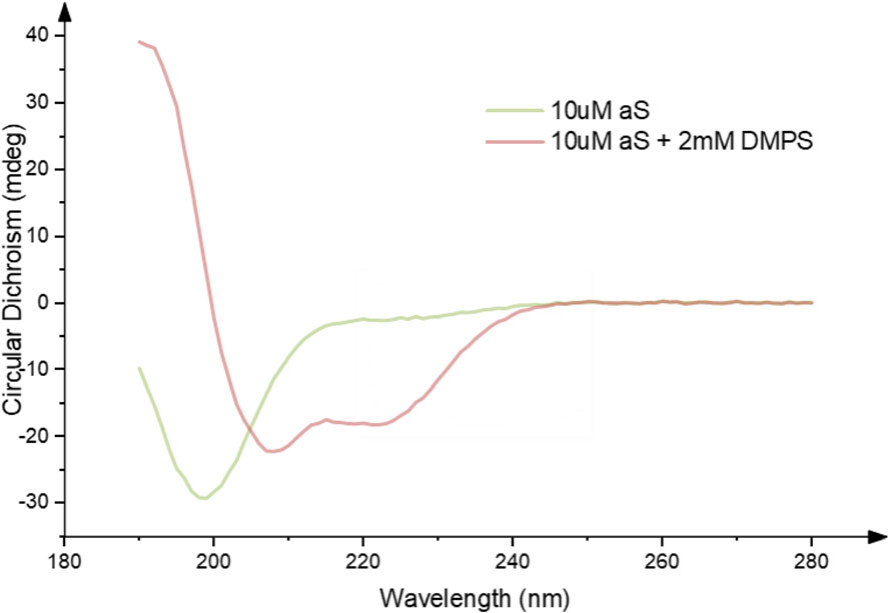
Change in Circular Dichroism (CD) signal in the far UV caused by the binding of αS to an excess of DMPS vesicles. This demonstrates a shift from a random coil structure in the absence of lipid vesicles (green), towards an alpha-helical secondary structure in the presence of DMPS lipid vesicles (red). Meade *et. al.* unpublished data reproducing data from Galvagnion et al. [34]

Despite considerable effort, the precise native structure of αS is still poorly defined. It has been variously described as intrinsically disordered [28, 29], helical [30, 31], or a combination of the two [32]. A helix-rich structure has been shown to be more readily populated in the presence of phospholipid membranes [33, 34] (Fig. 1), offering one possible insight towards the functional role of the protein.

Identifying the precise native state(s) of αS has certainly been hampered by the lack of knowledge of a clear function for the protein, its binding partners, or specific post-translational modifications (see below). The majority of studies have failed to take these variables into account. A wide range of publications have sought to interrogate the structure in a variety of different buffer conditions, including variations in salt, pH and lipid composition [35]. More recently, others have studied different modifications to the protein composition (e.g. phosphorylation, glycation, glycosylation, acetylation) and possible effects on protein structure and function [29, 36, 37]. Some groups have studied protein expression and aggregation in disease-relevant mammalian model systems to identify and understand possible roles for PTMs and the local environment on pathology.

A current consensus is that αS functions to promote membrane curvature, thereby contributing to synaptic trafficking and vesicle budding [38, 39]. This may be important given the association of αS with presynaptic terminal SNARE complexes [40], and suggests a potential role for αS in modulating dopamine release. This in turn has led to a number of studies investigating the transmission of the protein via synaptic terminals. Additional evidence lends support to a ‘prion-like’ hypothesis, whereby oligomeric αS can migrate between neurons to propagate formation of Lewy bodies throughout the *substantia nigra* and into extranigral regions. In particular, Bartels et al [30] and Wang et al [31] independently provided evidence that αS is able to fold into a stable helical structure by associating to form homotetrameric structures. This result was controversial as it was difficult to reproduce in vitro since multimers can disassemble upon cell lysis to generate aggregation prone monomers [41]. Later, others have reported that the structure could be recapitulated by the addition of lipids [42], providing helical multimers and evidence towards a native role for αS association in membrane interactions and in particular, vesicle budding. A similar effect has been observed either via N-terminal acetylation [43] or by extension of the N-terminus by 10 amino acids [31, 44], leading to formation of a persistent tetramer even in the absence of lipids [30]. Modifications to the N-terminus are known to be particularly important in driving folding towards a helical form of αS [31], which then impacts upon downstream aggregation [45].

Interestingly, a similar homotetrameric model for amyloidogenesis as a general principle had been proposed earlier [46, 47] based on the observed properties of a synthetic homotetramer formed from 4 equivalents of a short Glu/Gln rich peptide deliberately assembled in parallel on an artificial scaffold. In these experiments the peptide became significantly more α-helical and indefinitely stable at pH 7 when brought together in a parallel alignment, forming a homotetrameric arrangement. However, acidification transformed the α-helical aggregate, via a more elongated 4(3_10_) helix bundle [47] that led to tetramer aggregation, *en-route* to further elongation into four β-strands, seeding β-sheet aggregation and oligomerisation into matted amyloid-like fibrils. The key finding was that the tetrameric α-helix bundle was stabilised in water due to its hydrophobic core and polar hydrophilic exterior, like most proteins. However, the α-helix is in equilibrium with its more elongated 3_10_ helix analogue, and transition to a 4(3_10_)-helix bundle proceeds under acidosis conditions due to protonation of hydrophilic residues (Glu). Rearrangement of polar Glu/Gln residues to the interior of the helix core and some hydrophobic residues (Leu) to the exterior surface promotes aggregation. This led to core destabilization and an α-helix to 4(3_10_)-helix transition driven by inter-coil hydrogen bonds formed between facially paired protonated Glu residues (carboxylic acid dimers) and paired Gln residues (hydrogen bonded carboxamides). These interactions provided the catalyst for driving the equilibrium towards thermodynamically more stable strand/sheet formation and aggregation into oligomeric amyloids. For that particular peptide sequence, the process could be completely reversed back to the stable α-helical tetramers by restoring the pH to 7. Interestingly, acidosis has been associated with accumulation of αS oligomers [48, 49]. Local acidosis occurs at sites of inflammation and under conditions of metabolic stress (glycolysis and lactic acidosis), but whether this amyloidogenesis model with partial glutamate protonation or interstrand coupling of polar sidechains is relevant to αS oligomerisation and PD is unknown.

The current paradigm is that αS is likely to exist in vivo as an equilibrium mixture of unstructured monomer and statistically disfavoured helical oligomer(s), perhaps partially folded at membranes through phospholipid interactions. The alpha helical form of the protein may be required for an unknown native function but is not anticipated to be pathogenic, leading to the idea of stabilizing helical αS as a novel intervention strategy for PD. This might be similar to an approach used by Kelly and co-workers in stabilizing the native transthyretin fold, albeit targeting the protein with small molecules [50].

### α-Synuclein Misfolding: implications for PD

Following the implication of the SNCA gene, and therefore αS, as a leading cause of pathology in familial forms of PD (see below) [20–22], it was also shown to be the primary protein found within Lewy bodies [19]. In particular, a central hydrophobic region of the protein corresponding to residues 71–82 was found to be essential for the misfolding and aggregation of αS into fibrils. The 71–82 region was also found to be able to aggregate in isolation [51], its deletion (residues 71–82 [51] or 66–74 [52]) preventing aggregation of the protein and implicating these as key regions in misfolding and possibly instigation of amyloidosis. More recently, Tuttle et al. employed ssNMR to demonstrate that the structure of αS in its fibrilar β-sheet arrangement adopts a serpentine Greek key topology [24]. This structure again highlighted the importance of the 71–82 region in stabilizing the pathogenic conformation of αS, but importantly also highlighted a second critical region that is strongly associated with early onset mutations (in particular E46K, H50Q, A53T/E/V and G51D – see below). The region, spanning residues 45–57 is key in mediating β-strand to β-strand interactions in the fibril conformation. This also reflected an exposed surface on fibrils between residues 46–57, suggesting that this region of αS is accessible in the fibril (see below). More recently, a number of cryoEM structures of mature fibrilar forms of the protein has been solved by two independent research groups [25–27, 53] with many similarities to the ssNMR structure. Two structures display a Greek-key topology, with a further two characterised by a hydrophobic cleft stabilised by intermolecular salt bridges and additional interactions between the NAC and the N-terminus [53] (see below). In all cryoEM structures the fibrils form dimeric strands, with rotational symmetry about the axis. In the former two structure is provided by the seemingly exposed 45–57 region of the fibrillised protein. This region may therefore act as a hydrophobic ‘steric zipper’, as first described in amyloid fibrils by Eisenberg and colleagues [54], between adjacent protofibrils that then serves to facilitate the formation of a more mature double stranded fibril structure [25, 55].

### Genetic evidence for αS in PD

A relationship between genetics and PD was first identified in 1990, when members of an Italian-American family (the Contursi Kindred) were found to manifest inherited early onset PD. Studies subsequently found Lewy body pathology after autopsy [21] and the causative mutation leading to familial early on-set PD was located in the αS gene (SNCA) on chromosome four [20]. The specific mutation was an autosomal-dominant single base pair change in SNCA leading to the A53T substitution in αS [20]. Following this discovery, further autosomal dominant mutations in the SNCA gene have been found to cause familial PD. These include E46K [56–58], H50Q [59–62], G51D [59, 63], A53T [20, 64], A53E [65], A53V [66] and A30P [67–69] (Table 1). The most potent of known mutations, leading to the earliest onsets of the disease, is G51D. Interestingly, despite all of these single amino acid changes leading to early onset of PD, each provides very different effects on the αS aggregation rate and the oligomers that become populated. For instance, the E46K [56–58], H50Q [59–62] and A53T [20, 64] mutations all lead to an increased rate of fibril formation, whereas the G51D [69], A30P [67] and A53E [70] mutations appear to slow the rate of fibril formation. All mutations must therefore lead to either an increase in the aggregation rate, or a change in the oligomeric state or conformation that is populated upon aggregation, as well as a decrease in the normal tetramer:monomer ratios that facilitates these changes. The mutants collectively provide compelling evidence that aggregation of αS directly leads to early onset PD, while others more specifically provide indirect evidence that prefibrilar oligomers are more toxic than mature aggregated fibrils. In addition to changes in aggregation kinetics of mutant αS variants, differences in their association with phospholipid membranes have also been observed. Mutations typically result in reduced phospholipid binding, as for example in G51D, A30P [68, 69] and A53E [70] variants. In contrast E46K and A53T lead to increased phospholipid binding [58]. These observations suggest a functional relationship between αS and lipid binding that can become compromised by changes in interaction and structure in early onset mutants. In addition to missense mutations described above, autosomal dominant familial PD has been observed when the SNCA gene becomes duplicated or triplicated [71, 72]. Consistent with the role of αS in PD, examples where triplication has occurred have led to more severe forms of PD than in instances of gene duplication. This highlights the importance of intracellular concentrations in driving increased likelihood of αS misfolding, seeding, and ultimately to an early onset of the disease phenotype relative to sporadic cases of PD.

**Table 1:**
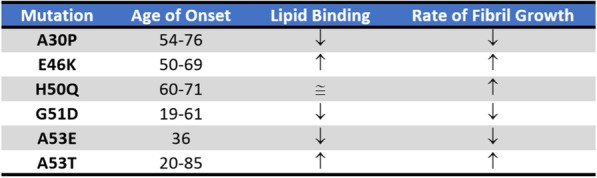
Comparison of the effects of age of onset [106], lipid binding [69, 70, 106] and fibril growth rates [69, 70, 106] of the different early onset mutations in the SNCA gene as compared to the wild-type protein. An additional mutation A53V has also been reported [66]. The mutations listed have additionally been described as leading to different clinical and pathological features [107]

### Fibril structure and early onset mutants

Recently, atomic resolution structures of the β-sheet rich fibrilar forms of αS have been elucidated. This was first reported by Tuttle et al [24] using an ssNMR approach to achieve 4.8 Å structural resolution (PDB ID 2n0a), and more recently by Guerrero et al [25] using cryoEM to obtain a 3.4 Å resolution structure (since named polymorph 1a; PDB ID 6h6b), closely followed by Li et al [26] to 3.1 Å resolution (polymorph 1a; PDB ID 6a6b). The deduced ‘Greek key’ conformation elucidated independently by all three groups is strikingly similar, showing that each αS subunit in the fibril adopts a β-sheet conformation, with hydrogen bonding between adjacent αS subunits, spaced 4.8–4.9 Å apart. The central β-sheet rich core of the structures is located between residues 42–102 and is comprised of an inner hydrophobic region of αS that interlocks into right-angled spirals. N-terminal residues 1–41 and C-terminal residues 103–121 display a flexible random coil arrangement that is consequently poorly resolved within the structure. The outer surface of the fibril is mostly hydrophilic, with the exception of two main regions; L38/V40 and F94/V95, with V82 providing further hydrophobicity [25, 26]. Moreover, the central hydrophobic region is comprised of Ala/Val residues, and one Ile [25]. All structures highlight a potential salt bridge between E46 and K80, which likely serves to stabilize the conformation. An additional protofibril structure known as polymorph 1b (PDB ID 6cu8) shares the kernel structure that comprises the dimeric protofilament, but differs in the interface packing (see section ‘Polymorphic amyloids - Rods and Twisters’ for more).

**Fig. 2:**
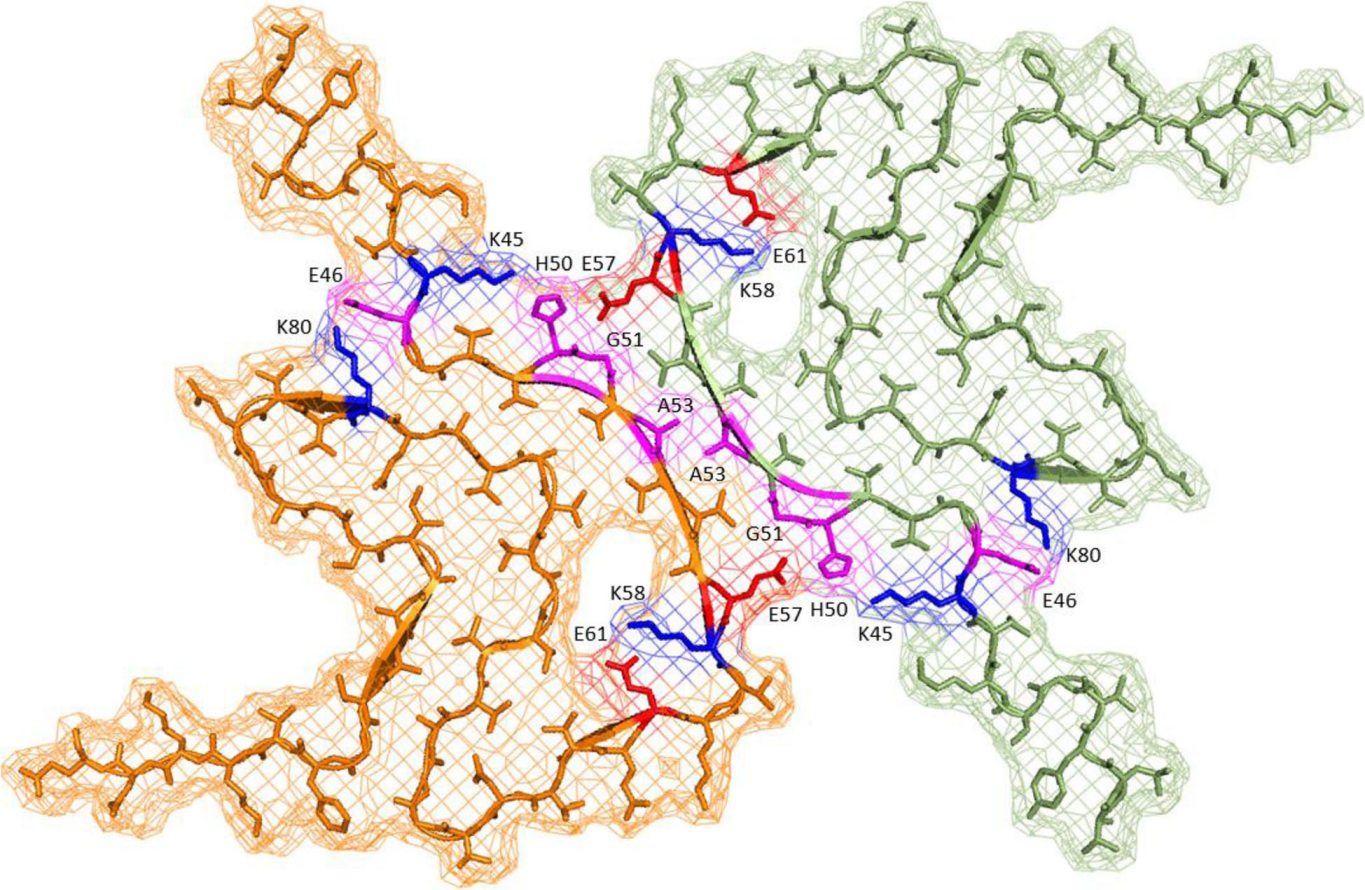
Structure of a single layer within a mature αS fibril. Based on the CryoEM structure published by Li et al [26] displaying formation of the ‘Greek Key’ topology with rotational symmetry about the axis of the fibril. The early onset mutations (E46K, H50Q, G51D/E, A53T) are highlighted (pink) in addition to three key electrostatic interactions that are perturbed in early onset PD (K58-E61, E46-K80 and K45/H50-E57)

Although both polymorph type 1a cryoEM structures exhibit many common features, the most notable is that the fibrils are 10 nm wide and composed of two adjacent protofilaments (Fig. 2). These interact to form a hydrophobic steric zipper, with a potential salt bridge being formed between E57 and H50 of the adjacent subunits. In contrast, the structure determined by ssNMR generated single strand fibrils with a width of 5 nm. It is therefore plausible that native αS can exist either as a single 5 nm protofilament, or as a dimerized 10 nm filament with rotational symmetry about the interface. Indeed, both structures have been observed in PD samples extracted from the *substantia nigra* [73]. The dimeric 10 nm filament is therefore likely to be a more mature fibril than the single 5 nm protofilament. This may also explain other structural discrepancies observed in amino acid orientations, which may be due to a more ordered energetically stable conformation upon dimerization of the protofilament.

There are a number of other differences between the two “polymorph 1a” cryoEM structures. For instance, in the cryoEM structure reported by Li et al [26], there is an additional salt bridge formed between residues E61 and K58 and this was not observed in the structure reported by Guerrero et al [25]. Instead, residue K58 is twisted towards the opposite side of the peptide backbone creating a cluster of positively charged residues (K43, K45, K58, H50) that provide excess electron density in this region. This was also not observed in the Tuttle et al ssNMR structure [24]. These differences could be caused by structural polymorphisms in this region between fibres, as a result of recombinant expression where PTMs are lacking or, as has been suggested [3], by an artefact in the Guerrero-Ferreira et al structure in which the construct used was a C-terminally truncated αS (1–121). However, for both cryoEM structures the fibres otherwise exhibit very similar overall topology and early onset residues display much the same interaction patterns.

The recently elucidated αS fibril structures are shedding new light on the mechanisms through which point mutations lead to early onset PD. A striking feature (Fig. 2) is that the fibril polymorph 1a interface is composed of small shallow hydrophobic residues (G51, A53, V55), that are flanked by strong ‘ionic locks’ (K45/H50➔E57). The β-sheet structure of each subunit is further stabilised by the existence of two further ionic locks, K58➔E61 and E46➔K80. Importantly, these electrostatic contacts are buried within the fibril core, away from the aqueous environment, potentially enhancing their energetic contribution to overall fibril stability. The individual mutations are discussed in detail below, each appearing to share the fundamental property of destabilizing the formation of mature fibrils (Fig. 2) and potentially increasing the duration that toxic oligomeric species remain stable within the cells.

#### E46K

In the majority of the structures the close proximity of E46 and K80 side chains suggest the formation of a stabilizing salt bridge [27] (Fig. 2). This salt bridge is compromised in the E46K [57] αS mutant, leading to electrostatic repulsion that destabilises the Greek key conformation and likely leads to an increased concentration of smaller oligomers rather than mature fibrils.

#### H50Q

As is the case for E46K, a similar explanation is offered for the mutation H50Q [61]. In both instances the interface between the protofibril dimers is destabilized, leading to a potential shift in the equilibrium towards smaller oligomers. Substituting His with Gln removes the positive charge on the imidazole at physiological pH, preventing formation of a stabilizing salt bridge with E57 on the adjacent filament, and also removes a potential intramolecular salt bridge with K45 that stabilises the Greek key formation. Although not observed in Nature, the E57K mutant [74] generates the same effect by mutagenesis of the partnering side-chain. This perturbation has been shown to lead to the formation of small oligomers that are highly toxic in animal models relative to αS mutants that display significantly enhanced aggregation rates [74].

#### G51D

Similarly, the G51D substitutions [63] on the neighbouring residue may inhibit fibril formation by loss of flexibility and hydrophobicity. It also introduces more steric bulk into the edge of the steric zipper region at the dimeric fibril interface (the G51 sidechain inserts between V55 and E57 on the opposing chain) as well as a potential charge repulsion with E57. This offers a potential explanation as to why this is the most potent of the known early onset mutations. It is also the slowest to aggregate in ThT experiments measuring fibril growth [69], supporting the hypothesis that increased lifetime of smaller oligomers can impart toxic effects. Moreover, the larger D residue is likely to sterically hinder the close interaction of the hydrophobic zipper and decrease local hydrophilicity, potentially inhibiting the formation of the dimeric protofibril. However, the structure from Li and co-workers suggests that a G51D change could impart an intramolecular attraction with K58 (Fig. 2).

#### A53E/T/V

A similar inhibition of the hydrophobic interaction between the two protofibrils can explain the early onset mutations based around A53. The A53T [20] and A53E [65] mutant side-chains are larger and more hydrophilic, and may again inhibit close contact and therefore hydrophobic zipper formation between adjoining protofibrils (A53 inserts between A53 and V55 side chains on the opposing chain). These changes therefore likely weaken hydrophobic packing within the steric zipper. On the contrary, the A53V [66] mutation would increase the hydrophobic interaction, but is a bulkier side chain and so may cause steric hindrance along the tightly intercalated steric zipper, thereby decreasing the contact between the involved residues.

#### A30P

The A30P [67] mutation occurs in the N-terminal random coil region, upstream of the hydrophobic Greek-key region, suggesting an alternative mechanism to toxicity. For example, the change may result in a compromised role in the native fold, possibly directly affecting interaction with phospholipid membranes.

### Summary

To summarize, early onset mutants found at H50, G51 and A53 can be rationalised by the cryoEM structures [25, 26] in a way that is not suggested by the earlier ssNMR structure [24]. In the former they appear to cause steric hindrance of the hydrophobic zipper interface formed between the two protofibrils. In the mature dimeric filaments, the proximity of the H50 and E57 side chains in adjacent protofibrils suggests a stabilizing salt bridge between protofibril subunits.

As for all types of amyloid, the aggregation of αS into mature fibrils may be a neuroprotective measure to shift the equilibrium away from soluble oligomers in a bid to reduce toxicity by lowering the number of exposed β-strands that present and can induce further aggregation. Therefore, when the formation of toxic oligomers is inhibited, this may block the formation of fibrils. On the other hand, if fibril formation is inhibited, this may have the counterproductive effect of serving to breakdown fibrils such that the toxic oligomers have longer lifetimes. One possibility is that the dimeric interface between the two protofibrils might function as a hinge point. Stabilization of the steric zipper leads to more mature fibrils that are less toxic, while mutations that weaken the interface (i.e H50Q, G51D and A53T/V/E, and the artificial mutation E57K) may lead to a population of more toxic smaller and therefore more soluble oligomers. There are most likely additional conformers that are yet to be elucidated, which may play important roles in the neurotoxicity of αS. Further experimentation is required to elucidate: ***i)*** the identity of oligomers of αS that are soluble and mobile versus insoluble and prone to fibrilisation, ***ii)*** the types of conformer within each oligomer population and how they are altered by mutations, ***iii)*** structure-function differences between oligomer populations. Addressing these points can distinguish those conformations that are most populated versus those most responsible for αS toxicity.

### Polymorphic amyloids - rods and twisters

**Fig. 3:**
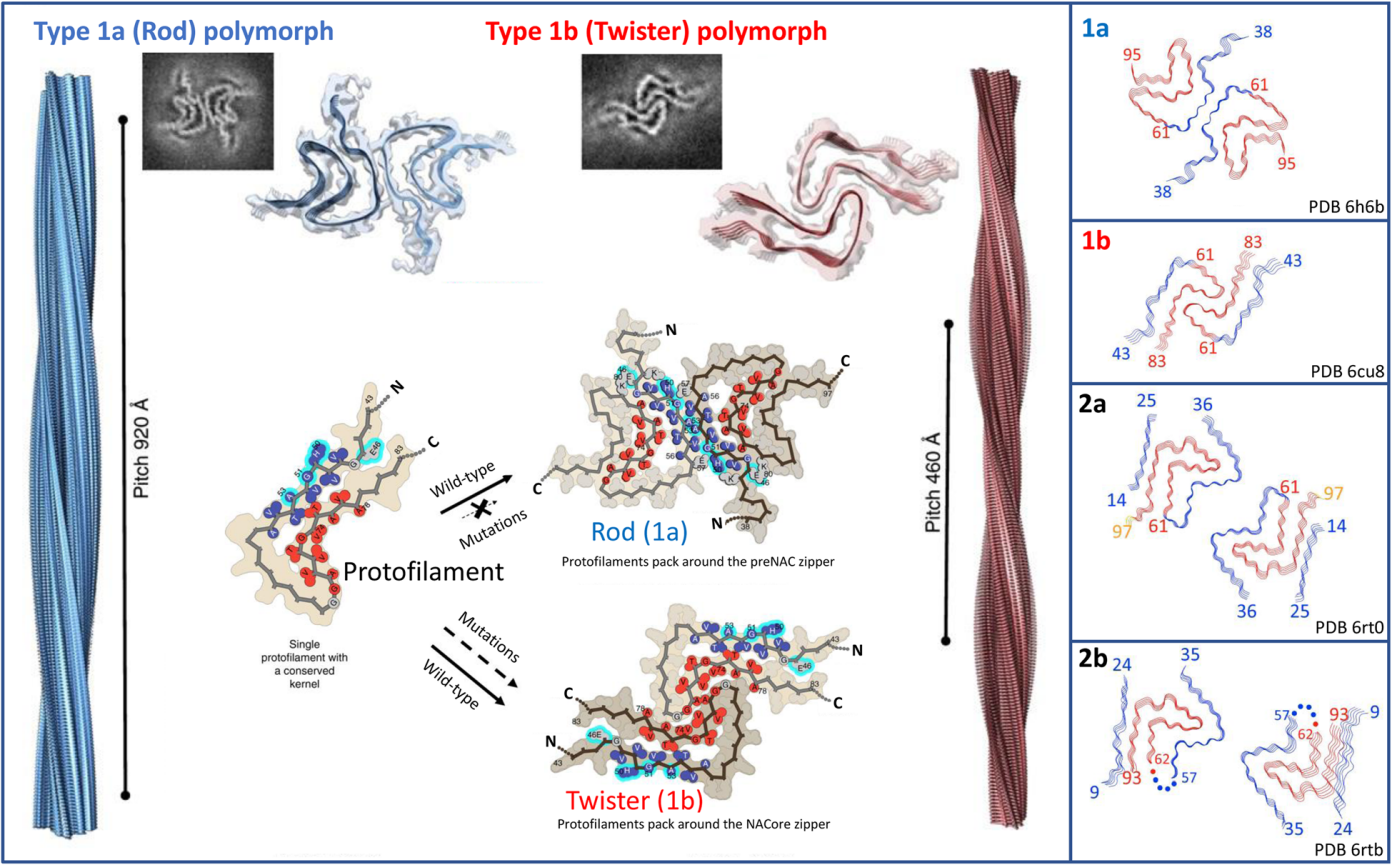
CryoEM structures of four distinct types of full length αS fibril. The four structures are known as type 1a ‘rod’ [25, 26], type 1b ‘twister’ [27], type 2a and type 2b polymorphs [53]. Single layer density slices within the rod structure have revealed a Greek Key topology with rotational symmetry about the axis of the fibril. In contrast, single layers within the twister structure reveal a β-arch motif. Both type 1 polymorphs contain two protofilaments composed of stacked β-sheets with rotational symmetry about the fibril axis. In contrast, type 2 polymorphs lack the steric zipper geometry identified in type 1 polymorphs and are instead characterised by a hydrophobic cleft that is stabilised by intermolecular salt bridges and additional interactions between the NAC and the N-terminus. Left Box) Shown is the 3D model of the type 1a (rod) and type 1b (twister) fibril polymorphs, respectively, with their distinctively different helical pitches depicted. Top) Shown are representative regions of density maps of both polymorphs are superimposed with their models showing match of side chains with cryoEM densities. Bottom) How a 5 nm protofilament [24] may represent a shared fibril kernel from which both rod and twister fibrils can develop. In rod fibrils the interface is composed of residues within the preNAC region (blue, residues 47–56), an area in which most of the early onset PD mutations are located (cyan). In the twister fibrils the interface is composed of residues within the NAC core region (red, residues 68–78). This suggests that early onset mutations disfavour the rod like fibrils over the twister structures, offering the possibility for fibril morphogenesis and the potential to shift the aS population towards a more toxic polymorph. The left hand panel has been adapted from Li et al. 2018 [27] (CC-BY 4.0). The right hand panels are adapted from Guerrero-Ferreira et al 2019 [53] (CC-BY-NC-ND 4.0) and show schematic representations of all four currently characterized αS polymorphs with the with the N-terminus in blue, the NAC region in red and the C-terminus in yellow

Following previous work there has very recently been described two additional αS polymorphic structures, named polymorph 2a (PDB ID 6rt0) and polymorph 2b (PDB ID 6rtb), both solved via cryoEM at 3.1 Å and 3.5 Å resolution respectively [53]. As for type 1 polymorphs these are composed of two protofilaments of 10 nm diameter but display very different arrangements (Fig. 3). In particular the steric zipper is missing in the type 2 polymorphs, which instead interact via K45-E57 (polymorph 2a) or K45-E46 (polymorph 2b) intermolecular salt bridges. In both polymorph 2 structures the steric zipper where familial mutation sites are found in polymorphs 1a-b, is replaced by a hydrophobic cleft. Moreover, in both structures the NAC region as before is buried but now additionally interacts with N-terminus of αS (via the C-terminal portion of NAC) that was not observed in polymorphs 1a-b.

Recently Li and co-workers used cryoEM to discern between two distinct types of mature polymorph 1 type fibre arrangements [27]. Both are 10 nm in width and bear many similarities to the earlier structures reported [24–26]. The single protofilament structure of 5 nm [24] resembles the common protofilament kernel of a bent β-arch that is found in both fibrils, suggesting this protofilament could be a precursor structure that gives rise to other types of polymorph in addition to the two reported. Of the two polymorphs observed, major differences in packing gave rise to structures described as ‘rods’ (protofilament polymorph type 1a) and ‘twisters’ (protofilament polymorph type 1b). There are two major differences between these subtypes. The fibre-pitch in the twister structures is 460 Å compared to 920 Å for the rods. The second key difference is the structure of each αS molecule within a given polymorph. In the twister structure each molecule forms a bent β-arch with a NACore interface (residues 68–78), whereas for the rod structure the bent β-arch contains additional ordered residues that lead to the formation of a ‘Greek-key’ fold as reported by others [24–26] with a preNAC interface (residues 47–56). In this work, of particular note is that fact that mutations associated with early-onset PD are located in the preNAC region. These would appear to disrupt the intermolecular interface of the rod structures, but not the interface of of the twister structures (see Fig. 3). This suggests that in cases of early onset PD the equilibrium might shift towards a higher amount of twister-like structures. This in turn implies that twister polymorphs, rather than rod polymorphs, may be the more disease-relevant of the two type 1 polymorphic species in contributing to PD pathology. Consistent with this recent structural evidence is the fact that others have also described distinct polymoph subtypes, including αS fibrils isolated from PD patient brains with distinct polymorphic structures with fibril widths of 5 and 10 nm respectively [73]. The increasing number of different strains identified may also account for distinct clinico-pathological characteristics within different synucleinopathies [75].

### Toxic versus non-toxic oligomeric conformations

The misfolding of soluble monomeric αS into insoluble fibrils observed in Lewy bodies requires the protein to exit from the usual folding trajectory. As molecules pass through a number of different transiently stable intermediate structures, there is the possibility for them to self-associate into oligomers via formation of β-strand to β-strand intermediates. It is gradually being accepted in the field that one or more specific, likely smaller, water soluble oligomers and their conformers are likely to represent the toxic species leading to disease [76, 77], directing research into identifying and characterizing these different oligomeric states and their relative toxicities. However, since these systems are metastable they are in constant flux and the oligomers are only transiently populated. Experiments that can delineate the precise states, structures and relative toxicities are therefore extremely challenging. However, in the last few years some inroads are finally beginning to be made.

Chen et al recently described a method for isolating stable toxic αS oligomers that have accumulated during amyloid formation, then characterized their structures [77]. They found two distinct subgroups of large stable oligomers, which they termed 10S and 15S oligomers, corresponding to an average of 18 and 29 monomer units per oligomer respectively. They were shown to be able to induce a toxic response at concentrations as low as 40 nM [77]. Atomic force microscopy (AFM) demonstrated that the oligomeric species were spherical in nature and contained ~ 35% β-sheet structure content with an antiparallel arrangement [77, 78], whereas more mature fibrils contained ~ 65% β-sheet structure [77] and are typically packed in a parallel arrangement [24, 25]. Further analysis by cryoEM demonstrated that both subgroups exist as either doughnut-like or cylindrical conformers, and in similar proportions. They displayed a hollow core with an increased solvent-exposed hydrophobic surface, suggesting that they may interact favourably with hydrophobic membranes. The pore-like structure has previously been proposed as an important feature for amyloid oligomers in general to impart their toxicity [79]. It is worth noting that they found that αS oligomers smaller than 14 αS molecules were unstable and dissociated into monomers. There is a strong possibility that some smaller, less stable, oligomers could represent the more toxic species, and further approaches are required to identify these and to discern their unique structural and biochemical properties.

Later, Fusco et al formed two distinct groups of αS oligomers, described as type-A and type-B [80]. Type-A were prepared in the presence of (-)-epigallocatechin-3-gallate (EGCG) and were deemed non-toxic species, while type-B were found to be similar to those observed by Chen et al. [77] and conferred cytotoxicity. The main difference between the two subspecies was their ability to disrupt lipid bilayers, with type-B conferring > 10 times the amount of disruption of type A, monomeric αS or even mature fibrils. One possibility is that type-B oligomers function by reducing mitochondrial activity in susceptible neurons [80]. This result has been further advanced by Ludtmann et al*,* who have demonstrated that αS oligomers can induce mitochondrial dysfunction by binding to the outer membrane and inhibiting ATP synthase by oxidation of the β-subunit [81]. Further aggregation of the oligomers can lead to increased ROS-induced opening of the mitochondrial permeability transition pore (PTP), leading to release of NADH into the cytosol [81], and depolarization of the mitochondrial membrane. These findings are supported by rat models in which the A30P mutation was also observed to cause mitochondrial impairment [82].

Klenerman and colleagues have been investigating the nature of different oligomeric species using Single-Molecule Förster Resonance Energy Transfer (FRET) Measurements. They have determined two distinct sub-populations of oligomers, termed Low-FRET and High-FRET [83], which appear to correlate with the Type-A and Type-B oligomers prepared by Fusco et al [80]*.* Their experiments suggest that formation of fibrils from monomeric αS follow a structured funnelling down the energy landscape, whereby monomeric αS first forms low-FRET, proteinase K sensitive, ThT inactive [84] oligomers with a diverse range of surface hydrophobicity [84] averaging 60 ± 2.6 nm in size. They then undergo a conversion step to a more compact, High-FRET, proteinase K resistant, cytotoxic, β-sheet rich, ThT active oligomer with a highly hydrophobic surface [84], averaging 80 ± 5.5 nm. These in turn form into the β-sheet rich, ThT active, less hydrophobic fibrils [83]. The High-FRET oligomers were found to be composed of 6–150 subunits, although the majority of species in the reaction were smaller than 10-mers [83], and found to be cytotoxic to primary neuronal cultures, by promoting ROS production [85]. Indeed ROS production was measured in cells exposed to concentrations of the high-FRET oligomers as low as 50 pM [83], a result that highlights the probability that this is the pathogenic form of αS.

Further delineating the pathogenic species, or alternatively better defining the native role and structure of αS, may provide *bone fide* targets or a viable approach for the rational design of drugs to prevent the death of susceptible neurons. Once the precise toxic species are determined, the design of drug candidates will become more rational and accelerated. Moreover, the experimental tools used to gain these insights may enable characterization of drug targets for other amyloidogeneic proteins involved in related age-related diseases, for which a similar amyloid assembly mechanism might prevail.

### Post-translational modifications

αS has been found to undergo a variety of post translational modifications (PTMs) in vivo, suggesting their importance in PD pathology. To date these have mainly included acetylation, phosphorylation and nitration which have been observed to affect the formation of different oligomers and fibril growth rates. N-terminal acetylation is a general post-translational modification of αS in mammalian cells [29]. It has been shown to cause increased helicity in the N-terminal region of the protein and decreased aggregation rates [86], as well as a 2-fold increase in affinity for lipid vesicles [87]. This PTM was included in the protein used by Li et al to identify the cryoEM structure of mature fibrils [26].

*Phosphorylation* has been identified on residues S87, Y125, Y133 and Y136 [37], but the most prominent pathologically relevant phosphorylation site appears to be on residue S129 [88]. This PTM has been found on more than 90% of the αS in Lewy bodies, but only 4% of the soluble αS, extracted from brain tissues of PD sufferers [89], and it has been shown to increase the rate of αS fibrilisation in vitro [88]. The specific function of this PTM remains unclear, with both toxic and protective effects reported in different animal and cell models, confounding the issue. Toxicity was accelerated in αS-overexpressing SH-SY5Y cells when phosphorylation of S129 was increased [90], and neuronal loss was observed to decrease in *Drosophila melanogaster* when S129 phosphorylation was inhibited [91]. The opposite has been found in yeast and rat studies where knockouts preventing S129 phosphorylation were observed to increase αS toxicity [92, 93], and formation of beta-sheet rich aggregates [93]. It therefore seems likely that this PTM plays a role in αS toxicity, but the nature of that role so far remains unclear and seems model dependent.

*Nitration*, caused by increasing levels of oxidative stress within neurons, is another commonly seen PTM of αS that has been detected on tyrosine residues Y39, Y125, Y133 and Y136 [36]. It is unclear whether this is caused by early stage pathogenesis of PD, or is a mitigating factor leading to PD. The most interesting, physiologically relevant, nitration appears to be at Y39, which has been observed to inhibit fibril formation and stabilize oligomeric species via dityrosine crosslinking [94]. It has been shown to inhibit αS association to synthetic vesicles [95], potentially mirroring the effects of the A30P [67] early onset mutation, adding further credibility to the idea of the toxic form of αS being an oligomer species rather than fibril. Dopaminergic neurons in the *substantia nigra* likely have a considerable energetic demand to support their unmyelinated axonal arbor [96] which results in the production of, and susceptibility to, reactive oxygen species [97], possibly explaining why these neurons are the first to become susceptible to αS toxicity.

Oxidative stress is seen to play a very important role in αS aggregation by affecting PTMs in the molecule, but it remains unclear whether they are a causative effect, which would open new targets for the treatment of PD, or a by-product further propagating a cascade effect of PD progression, explaining why the disease progresses so rapidly after its initial onset.

### Misfolding via a 3_10_ intermediate?

**Fig. 4:**
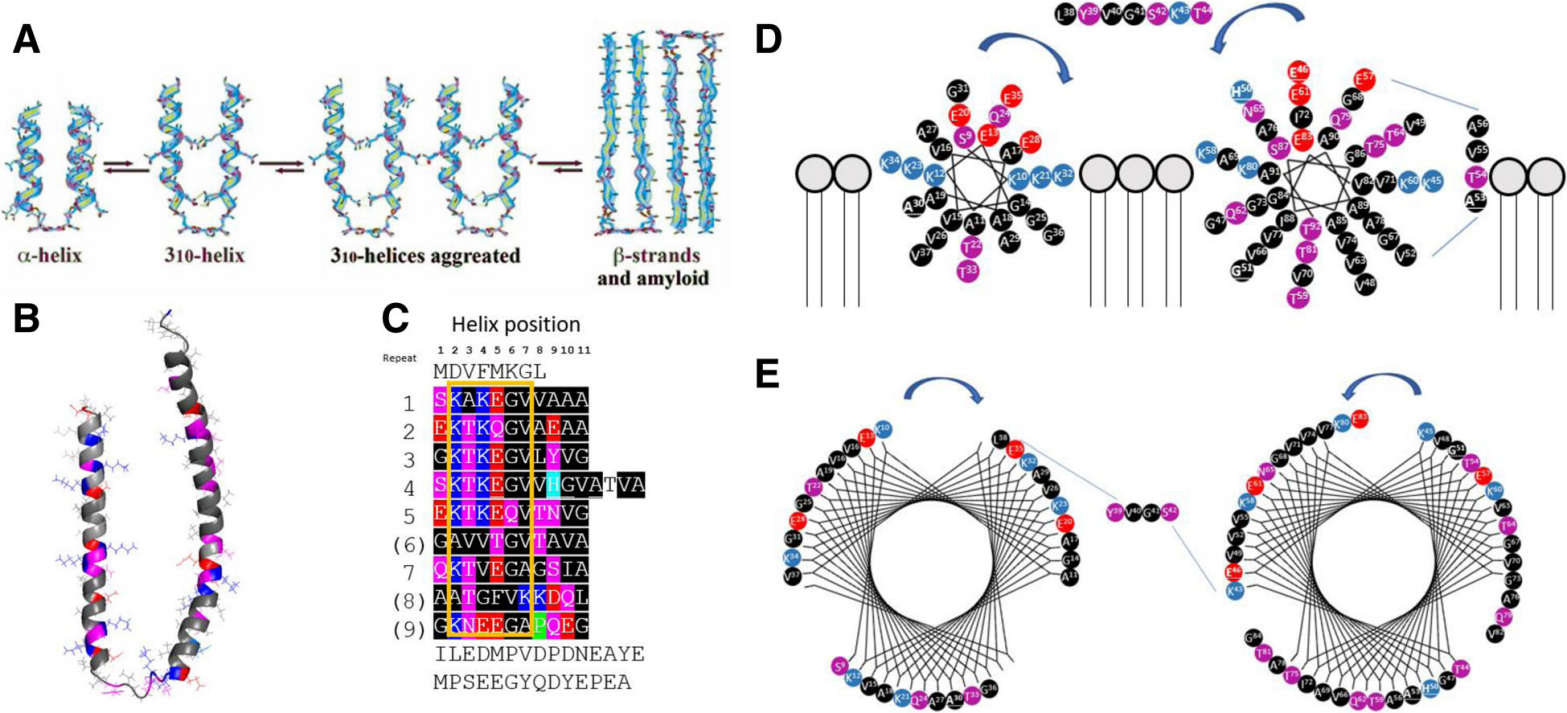
The KTKEGV imperfect repeats found within the αS structure. **a** Model of conformational transition proposed by Singh et al. [47] (CC BY-NC 4.0) of the transition of a 4-peptide bundle into amyloid fibrils, from an alpha-helix into a β-sheet fibril via aggregation induced stabilisation of anti-parallel 3_10_-helix bundles. This model may be representative of the transitions which occur with aS from an alpha–helix membrane bound monomer to β-sheet fibril. **b** Structure of the micelle bound human aS, published by Ulmer et al., determined by solution NMR spectroscopy [99], highlighting the antiparallel α-helices of the membrane bound αS monomer, helix 1 spanning from Val [3]-Val [38] and helix 2 spanning from Lys [46]-Thr [93], connected by a well ordered linker. **c** The linear 140 residues of human aS arranged into KTKEGV imperfect repeats 1–9. Blue = basic; light blue = his; red = acidic; purple = polar uncharged; black = nonpolar. **d** Shown is a colour coded schematic with repeats 1–7 arranged into two 11/3 helix (3 turns over 11 residues), adapted from the αS helical wheels proposed by Dettmar 2018 [100] and Bendor et al. 2013 [101] representative of the membrane induced amphipathic helix. It has been proposed that lysine rich positions (blue) interact with negatively charged lipid head groups, while hydrophobic regions (black, grey area) interact with membrane lipids. Interestingly the Gly residues are found at the hydrophobic-water boundaries of the core, and are found on the adjacent helix face, which may be important in facilitating alpha to β switching at the water membrane, as previously seen in amyloid beta [102]. The position of single amino acid changes associated with early onset PD mutations might destabilise sidechain-sidechain packing that promotes formation of the helix and thereby accelerate the pathway toward amyloidosis. **e** Proposed structure of 2 × 3_10_ helical wheel, formed by constriction of the α–helical domains seen in the micelle structure, clearly shows that the separation of the Lys and Glu residues in the aS amino acid sequence causes then to stack on top of each other stabilising the 3_10_ intermediate, driving the energetic landscape towards the β-sheet fibril. Most interesting here is that the the first of the ‘ionic locks’ observed in the cryoEM structures is already formed in this structure, between K58-E61. In this proposed structure there does not appear to be a membrane binding domain. Potentially this structural change from α-helix to 3_10_ intermediate could cause membrane disruption and mediate toxicity of αS

Could the same KTKEGV repeat sequences that are thought to stabilize the tetramer be involved in amyloid formation, or possibly act as a nucleation sequence via a 3_10_ intermediate? In the helical model shown [98] (Fig. 4d), there is evidence that specific amino acid types can become periodic within helical structures. This could explain interaction with lipids, multimerization into a tetrameric structure, and have implications for a functional role in vesicle budding and neurotransmitter release. Some early onset mutants may also shift equilibria and lead to loss of interactions within helical multimers and/or with the membrane leading to increased likelihood of aggregation. Indeed, KTKEGV repeat motifs have been speculated to be key mediators of normal αS tetramerization. Their mutation has been hypothesized to lead to formation of monomers as well as neurotoxicity [98]. Moreover, it has been shown that abrogating native αS tetramers in mice can lead to an L-DOPA-Responsive motor syndrome that closely resembles PD [103]. By introducing a series of E➔K mutations into several of the KTKEGV repeats it was shown that tetrameric αS formation could be abrogated therefore leading to an increased likelihood of misfolding [100].

An 11/3 helical wheel projection (Fig. 4d) implies an amphipathic helix with a hydrophobic face that can conceivably interact with lipids and a polar face that might interact with the solute. The two faces can be separated by a Lys rich seam that can potentially interact with negative charged head groups within the lipid [100]. There is likely to be an equilibrium between multimers and lipid binding in addition to changes in folded state structure. In addition, early onset mutations may play a dual playing in both destabilising the helical structures and their ability to interact with lipids, while destabilising rod polymorphs in favour of twisters.

Conformational transformation of natively folded αS into a partially folded intermediate (Fig. 4e) might account for aggregation and fibrillation. Given the potential helical structure of the native protein, particularly upon exposure to lipids or a membrane environment, one possibility is that the interactions which natively stabilise this structure are lost in aqueous environments. This might lead to destabilisation in favour of a β-sheet rich structure that is ultimately prone to aggregation and fibrilisation. One possibility is that a 3_10_ helix becomes populated en route from a compact α-helical structure stabilised by i➔i + 4 contacts to a more stretched helix stabilised by i➔i + 2 contacts. The latter 3_10_ helical structure might act as the first scaffold responsible for initiating further elongation to strands and sheets involved in early amyloid assembly events. In a 3_10_ helix model, the spacing of Lys and Glu residues in KTEGV repeats enables these residues to stack on top of one another (Fig. 4e), which we predict could stabilise such a misfolded intermediate structure over an α-helix, with this shift in equilibrium potentially leading to further elongation to β-strands that pair in β-sheets. Notably, the K58-E61 ‘ionic lock’ observed in the CryoEM ‘Greek-key’ structure is already in position in this model. This conformational change may tighten membrane bound helices and lead to disruption of lipid bilayers believed to be key for toxicity of toxic type-B oligomers [80].

### Diagnostics, therapies and Theranostics

Recent advances in our understanding of how αS confers its cytotoxic effects in susceptible neurons will invariably direct future avenues of study to the prevention and diagnosis of synucleinopathies. The focus in the field has previously been directed towards preventing the formation of fibrils, when in fact the toxic effects of the protein may occur much earlier at the soluble oligomer stage and be independent of fibril formation. The primary focus should be to understand the specific pre-fibril, soluble oligomer(s) of αS and their specific conformations, so that future treatments can be designed to prevent their formation or inhibit their interactions that mediate toxicity. This may represent a more promising approach to drug discovery. Such protein-protein interactions (PPIs) are often difficult to target with small molecule drugs, owing to the many points of contact needed over large, solvent-exposed, polar and shallow, surface areas in order to drive both affinity and selectivity of interaction. Consequently, larger biologics and brain-permeable peptidomimetics are increasingly becoming of interest for modulating polar PPIs as they are large enough to make multiple interactions, distinguish between conformations, or stabilize non-toxic oligomers. If the equilibrium could be shifted away from the toxic oligomers, this may permit the native functionality of αS to remain unaltered by treatment. A promising peptide is currently in development based on the region relating to the early onset mutations, between residues 45–54 [104].

Understanding the identity and properties of early stage soluble oligomers that enable identification of toxic oligomers could also enable the development of a diagnostic for early identification of risk of PD, allowing treatment to begin early to prevent disease. A molecule with high affinity and selectivity for the toxic conformation of αS could be used to show the extent of the disease progression also acting as a valuable biomarker to support drug discovery. Such approaches clearly need to be coupled with the added challenge of improved diagnostics that are able to detect dopaminergic loss and Lewy body accumulation in the years and decades before symptoms present. Drugs that are applied at such a later stage are unlikely to be able to reverse symptoms. They may also be too late to prevent further pathologies resulting from affected pathways downstream of neuronal damage and loss that has already been incurred.

### Future directions

As has been the case for other amyloidogenic proteins, a major advance in the field is likely to come from further structural studies from human brain samples [105]. This also needs to be coupled with more techniques that can interrogate transiently formed metastable species (ideally in conditions resembling the chemical complexity faced in neurons), and not only easily isolated endpoint products. As we have discussed, inroads towards these objectives are being made. Other questions remain and will surely be addressed over time. For example, which populations of αS have statistical weight? How can we best judge which strains are relevant? Which forms interact with lipids, and which (if any) with other compartments? Which αS strains can form such interactions and what are their affinities? Do different forms of αS co-assemble with other αS forms, or in time with other proteins? What are the relative stabilities of different αS assemblies and co-assemblies? Experiments aimed at addressing some of the above questions will help the community to embed and judge structural polymorphisms in a disease relevant context.

## Conclusions

Gaining a better understanding of αS structure, folding and function is complicated by the dynamic nature of the protein, which can form a range of monomeric and oligomeric species, different conformers that may be dependent upon the environment, different definitions of the native structure, posttranslational modifications and interactions with lipids or other agents in the neuronal environment. Moreover, fibrils grown under controlled experimental laboratory conditions understandably cannot mimic all (unknown) physiological conditions that may influence fibril development in the brain, where there may be important differences, including competing and dynamic events that may produce different oligomeric structures. All of these variables currently make the understanding of αS properties difficult to interpret both in its native and in diseased states. However, recent structural insights have begun to progress the understanding of structure, folding and function suggesting that rational approaches to a designed treatment for PD and other synucleinopathies are now closer than ever before.

## Data Availability

Not applicable.

## Abbreviations

DLB: Dementia with Lewy bodies
MSA: Multiple system atrophy
PD: Parkinson’s disease
SNCA: SyNuClein Alpha gene that codes for the αS protein
αS: Alpha-synuclein, the major constituent of Lewy bodies and a pathogenic hallmark of all synucleinopathathies
